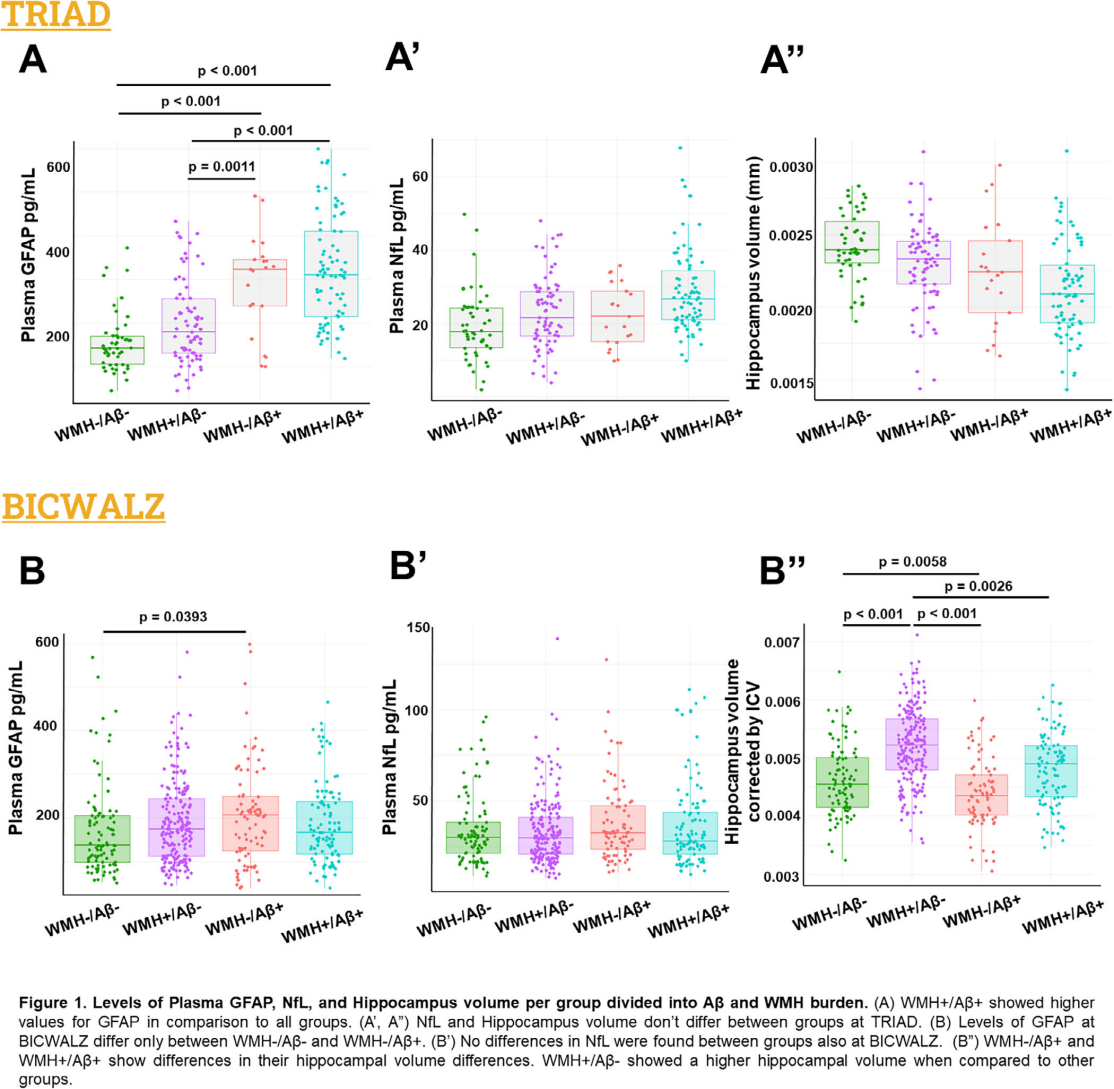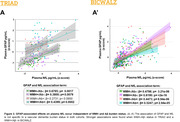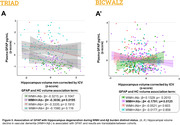# Hippocampal degeneration is associated with GFAP levels in individuals with vascular dementia independently of amyloidosis

**DOI:** 10.1002/alz.092720

**Published:** 2025-01-03

**Authors:** Markley Oliveira, Bruna Bellaver, Firoza Z Lussier, Pamela C.L. Ferreira, Guilherme Povala, Douglas Teixeira Leffa, Guilherme Bauer‐Negrini, Matheus Scarpatto Rodrigues, Sarah Abbas, Hussein Zalzale, Francieli Rohden, Cynthia Felix, Carolina Soares, Pampa Saha, Emma Patrice Ruppert, Vitor Hugo Machado, Andreia Silva da Rocha, Dana Tudorascu, Andrea L. Benedet, Nicholas J. Ashton, Cécile Tissot, Joseph Therriault, Stijn Servaes, Jenna Stevenson, Nesrine Rahmouni, Chang Hyung Hong, Hyun Woong Roh, Eduardo R. Zimmer, Kaj Blennow, Henrik Zetterberg, Thomas K Karikari, Pedro Rosa‐Neto, Sang Joon Son, Tharick A. Pascoal

**Affiliations:** ^1^ University of Pittsburgh, Pittsburgh, PA USA; ^2^ Department of Psychiatry, University of Pittsburgh School of Medicine, Pittsburgh, PA USA; ^3^ Department of Psychiatry and Neurochemistry, Institute of Neuroscience and Physiology, The Sahlgrenska Academy, University of Gothenburg, Mölndal, Gothenburg Sweden; ^4^ Department of Psychiatry and Neurochemistry, Institute of Neuroscience and Physiology, The Sahlgrenska Academy, University of Gothenburg, Mölndal Sweden; ^5^ Lawrence Berkeley National Laboratory, Berkeley, CA USA; ^6^ Translational Neuroimaging Laboratory, The McGill University Research Centre for Studies in Aging, Montréal, QC Canada; ^7^ Translational Neuroimaging Laboratory, The McGill University Research Centre for Studies in Aging, Montreal, QC Canada; ^8^ Ajou University School of Medicine, Suwon Korea, Republic of (South); ^9^ Ajou University School of Medicine, Suwon, Gyeonggido Korea, Republic of (South); ^10^ Universidade Federal do Rio Grande do Sul, Porto Alegre Brazil; ^11^ Brain Institute of Rio Grande Do Sul, PUCRS, Porto Alegre, RS Brazil; ^12^ McGill Centre for Studies in Aging, Montreal, QC Canada; ^13^ Institute of Neuroscience and Physiology, The Sahlgrenska Academy at the University of Gothenburg, Mölndal Sweden; ^14^ University of Wisconsin‐Madison, Madison, WI USA; ^15^ UK Dementia Research Institute at UCL, London United Kingdom; ^16^ Department of Psychiatry, School of Medicine, University of Pittsburgh, Pittsburgh, PA USA; ^17^ McGill University Research Centre for Studies in Aging, Montreal, QC Canada; ^18^ University of Pittsburgh School of Medicine, Pittsburgh, PA USA; ^19^ Departments of Psychiatry and Neurology, University of Pittsburgh School of Medicine, Pittsburgh, PA USA

## Abstract

**Background:**

Vascular cognitive impairment/dementia (VD) is the second most prevalent cause of dementia following Alzheimer’s disease (AD). VD is characterized by the progression of white matter hyperintensity burden (WMH) and associated neurodegeneration. GFAP, a biomarker for reactive astrogliosis, is associated with Aβ pathology and mediates tau‐pathology in preclinical AD. However, the association of GFAP levels with the markers associated with VD is poorly understood.

**Method:**

We assessed 796 participants from a research cohort (TRIAD: 355) and a memory clinic cohort (BICWALZ: 441) that were divided into four groups according to their Aβ (Aβ+/Aβ‐) and WMH status (WMH+/WMH‐). WMH values were corrected by intracranial volume, and cutoffs were determined based on the highest WMH value within 50% of the individuals with the lowest WMH rate in the CU Aβ‐ group. Biomarker mean level differences between groups were estimated via Ancova Tukey’s test, and linear regressions accounting for age, sex, and cognitive status were used to estimate the association.

**Result:**

For TRIAD, WMH+/Aβ+ individuals exhibit higher levels of plasma GFAP when compared to all groups except WMH‐/Aβ+, with no differences in NfL levels and hippocampal volume between groups (**Fig. 1A**). In BICWALZ, only hippocampal volume differs between groups with lower levels observed in both WMH‐/Aβ‐ and WMH‐/Aβ+ (**Fig. 1B’’**). The association of GFAP and NfL was observed in all groups (**Fig. 2A, 2B’**). On the other hand, hippocampal degeneration was associated with higher GFAP levels in individuals with abnormal WMH and absence of Aβ burden in both cohorts (**Fig. 3A**: TRIAD: β = ‐0.3036; p = 0.0195; **Fig. 3B’**: BICWALZ: β = ‐0.1791; p = 0.0125).

**Conclusion:**

Our findings suggest that astrocyte reactivity, as indicated by plasma GFAP levels, plays a significant role in the hippocampal atrophy observed in patients with vascular disease. These results suggest that therapies targeting astrocyte reactivity could potentially alleviate progressive cognitive deficits commonly found in patients with chronic vasculopathy.